# Do We Tell Our Coronary Patients Enough?

**Published:** 1986-10

**Authors:** Carol P. Trelawny-Ross, Stephen C. Jordan

**Affiliations:** From the Department of Mental Health, University of Bristol and the Cardiac Department of the Bristol Royal Infirmary; From the Department of Mental Health, University of Bristol and the Cardiac Department of the Bristol Royal Infirmary

## Abstract

Thirty two men and their wives discussed their experiences of the admission to hospital and subsequent rehabilitation following a threatened or confirmed myocardial infarction. Over all, the level of information and understanding about their condition was poor. Patients would have liked to have had more specific advice about rehabilitation and to have been told more about their condition. Thirteen discharging doctors indicated on a short questionnaire what advice they had given to their patients. This exercise confirmed the suspicion that while patients' memory might have been poor, they had not been given extensive information before they left hospital. In particular, although all of the men were married and under 65 years of age, only 38% of the doctors said that they had discussed sexual activity and of the whole sample (31 men) only 25% of the men recalled having been advised on this subject. Patients and their wives felt that they would have worried less if they had been better informed. Persistent symptoms were related more to worry and depression than to the extent of myocardial damage.


### Do we tell our coronary patients enough?

**Carol P. Trelawny-Ross, M.Sc.**
**Stephen C. Jordan, F.R.C.P.**
From the Department of Mental Health, University of Bristol and the Cardiac Department of the Bristol Royal Infirmary

### SUMMARY

Thirty two men and their wives discussed their experiences of the admission to hospital and subsequent rehabilitation following a threatened or confirmed myocardial infarction. Over all, the level of information and understanding about their condition was poor. Patients would have liked to have had more specific advice about rehabilitation and to have been told more about their condition. Thirteen discharging doctors indicated on a short questionnaire what advice they had given to their patients. This exercise confirmed the suspicion that while patients' memory might have been poor, they had not been given extensive information before they left hospital. In particular, although all of the men were married and under 65 years of age, only 38% of the doctors said that they had discussed sexual activity and of the whole sample (31 men) only 25% of the men recalled having been advised on this subject. Patients and their wives felt that they would have worried less if they had been better informed. Persistent symptoms were related more to worry and depression than to the extent of myocardial damage.

### INTRODUCTION

Patients have complained to us that they have not been told enough about their condition or given much useful advice about rehabilitation. Others have reported similar findings (1, 2). We felt that it would be useful to investigate the complaint of being poorly informed and to look at the possible consequences.

It has been shown that specific information and advice increases patients' understanding of their condition (3), can increase compliance (4), increases satisfaction with their treatment (5), reduces anxiety and depression (6), and does not increase side effects (7).

Several reasons have been suggested for patients' complaints of being inadequately informed or advised. Doctors may volunteer very little information (8). Information offered may be poorly understood and consequently is more likely to be forgotten or remembered in a distorted version. Patients are frequently reluctant to ask questions of busy doctors, and doctors may be reluctant to encourage them to do so (2). Even when given an opportunity to ask questions, it may be difficult for patients to anticipate what they are going to want to know once they have been discharged (9, 10) and even given an 'ideal' consultation, memory unaided by recall cues may be poor for many patients, especially if they are anxious or depressed. Above all, patients are often so worried about their immediate condition that they do not readily assimilate advice offered.

Correspondence to: Mrs C. P. Trelawny-Ross, Department of Mental Health, University of Bristol, 41 St Michael's Hill, Bristol BS2 8DZ.

In this study we have attempted to discover what doctors were advising patients and what patients remembered. We then considered the implications of these findings for the patients in the problems they reported and their outcome at six months.

### PATIENTS AND METHOD

Thirty one men who were admitted to a teaching hospital with a suspected myocardial infarction were each seen on four occasions in the course of six months. The men were all married and under sixty five years of age (range 32–64). The patients were all admitted as emergencies under consultant physicians to a coronary care unit, 80% of them spent up to three days there and between 6 and 19 days in a general medical ward and therefore could be considered relatively uncomplicated. The remaining 20% spent longer periods in hospital because of complications or persisting pain.

The severity of their heart attack was estimated by one of us (SCJ) using a modified Norris index (11, 12) (position of the infarct, systolic blood pressure on admission, radiology, previous infarcts) and in addition maximum enzyme levels and occurences of arrythmias. The maximum possible score was 20, the range of actual scores was 1 to 15.

The following information was sought at the interviews:

1. How much specific information or advice the patients had been given about smoking, exercise, diet, sexual activity, and driving.
2. Their general level of informedness and understanding was assessed on a 5 point scale. One was scored if the patient reported having discussed his condition and rehabilitation with a doctor or senior member of nursing staff and was able to show that he had a good understanding of both his present condition and his rehabilitation plans and expectations. Five was scored when the patient reported virtually no contact with medical or nursing staff providing information or advice, supported by a very poor understanding of his diagnosis or condition.
3. Patients were asked about the problems and worries they had experienced so far. A 'check list' based on Brown's survey of doctors treating patients with heart disease (13) was used (see Table 4). Patients were then asked if they had had any other problems or worries not mentioned on the list.
4. Outcome was assessed in terms of return to work, exercise, leisure activity, and return to normal sexual activity.
5. Compliance with advice to stop smoking was assessed.
6. Anxiety and depression were assessed using Goldberg's clinical interview schedule (14).

Ratings for 2 and 4 above, and a number of other potential contributors to outcome, e.g. somatic symptoms, weight, and General Practitioner support were developed from Goldberg's general rules for scoring.

For this part of the study t tests, Pearson product moment correlational and multiple regression analyses were used to assess significance. Details are being reported elsewhere (15).

**Table 1**

**Patient's memory of information and advice given prior to discharge**

| *Rating* | *n* | *%* |
|---|---|---|
| 1 (excellent) | 6 | 19 |
| 2 | 6 | 19 |
| 3 | 11 | 36 |
| 4 | 7 | 23 |
| 5 (v. poor) | 1 | 3 |
| Totals | 31 | 100 |

**Table 2**

**Support from General Practitioner**

| *Rating* | *n* | *%* |
|---|---|---|
| 1 (excellent) | 11 | 36 |
| 2 | 4 | 13 |
| 3 | 7 | 23 |
| 4 | 4 | 13 |
| 5 (v. poor) | 5 | 16 |
| Totals | 31 | 100 |

### RESULTS

The levels of 'informedness' are shown in Tables 1 and 3. Doctors' contributions are shown in Table 3 and may be inferred from Table 2, as part of the 'support' described by the patients was their doctors' willingness to explain and advise. The patients 'worries and problems' are shown in Table 4. No patients considered themselves to have been without problems of any kind. It can be seen that only 'money problems' are likely to be completely unrelated to levels of information and advice. Patients' and spouses' smoking patterns (Table 5) also reflect information and advice given. The correlations among variables associated with information, advice and outcome at 6 months are shown in Tables 7 and 8.

### DISCUSSION

The majority of the patients were not well informed and many had a poor understanding of their condition (Table 1), a few were mistaken in their beliefs about their diagnosis. Some had excellent support from their General Practitioners (Table 2) who may have provided the information and advice that the patients needed. On specific items of advice patients' memory was mixed but the recall rate among those whose doctors reported advising them was considerably better than for the whole sample (Table 3). Of particular note is the low level of advising and memory of advice about sexual activity.

All smoking patients were advised to stop, half of them reported having done so (t=4.939 p<.001); wives on the other hand did not change their habits (Table 5).

Lack of information and advice was seen by 18 (56%) of the patients to have contributed to their 'worries and problems' (Table 4).

**Table 3**

**Information and advice: patients' memory and doctors' reports (medical advice given)**

| | *Recorded by doctor* | | *Patients' recall* | | *All patients' recall* | |
|---|---|---|---|---|---|---|
| | *(n=13)* | | | | *(n=31)* | |
| | *n* | *%* | *n* | *%* | *n* | *%* |
| Smoking | 11 | 85 | 9 | 82 | 17 | 54 |
| Diet | 8 | 62 | 6 | 75 | 12 | 39 |
| Exercise | 13 | 100 | 7 | 54 | 14 | 45 |
| Sexual activity | 5 | 38 | 2 | 15 | 8 | 25 |
| Return to work | 13 | 100 | 8 | 62 | NA | |
| Driving the car | NA | | NA | | 10 | 32 |

**Table 4**

**Worries and problems associated with the 'heart attack' (data from the 2 month interview, n=32)**

| | *n* | *%* |
|---|---|---|
| Depression | 20 | 63 |
| Anxiety | 19 | 59 |
| Not knowing what can/can't do | 18 | 56 |
| Worry—long term expectations | 16 | 50 |
| Difficulty changing habits: Smoking | 10 | 31 |
| Diet | 4 | 18 |
| Drinking | 3 | 9 |
| Conflicting advice from doctors | 13 | 41 |
| Feeling useless | 13 | 41 |
| Restrictive wife | 13 | 41 |
| Being overprotected (family and friends) | 13 | 41 |
| 'Too ill' to consider returning to work | 10 | 31 |
| Money problems | 8 | 21 |
| Frequent visits to G.P. or Out-patients | 8 | 21 |
| Problems in resuming sexual activity | 7 (of 20) | 35 |
| Difficulty expressing worries to doctors | 5 | 16 |

Over 70% of the patients left hospital on prescribed medication and most of them continued with the same range of drugs throughout the six months (Table 6). Many of the men relied on their wives to dole out their pills. Others had 'had a go' to see how they managed without them. One man used his imagination to decide when and how many of his diuretics to take according to whether he was planning to have a light or a heavy drinking lunch. Several of the men who had been given glyceryl trinitrate to take as required said they rarely used it as the chest pain was preferable to the headaches they suffered when they used this drug. Few reported being given specific advice about the use of this or any other drug with the exception of warfarin. Many of the men were concerned about the side effects of their drugs. (It was not possible to gather data about the extent of compliance for the whole sample.)

### OUTCOME AT SIX MONTHS

Only just over half of the men could be rated as having returned to normal or optimal levels of activity by six months (Table 7). Several variables were found to have significant correlations with one or more aspects of outcome (Table 8).

In addition to the variables set out in Table 8, having 'symptoms' correlated significantly with how ill the patient rated himself r=0.785 (0.001), depression r=0.693 (p<0.001), with his smoking habits r=0.433 (p<0.001),

**Table 5**

**Patient and spouse smoking habits (number of cigarettes daily)**

| *Patient* | *On admission* *n* | *%* | *10 days* *n* | *%* | *2 months* *n* | *%* | *6 months* *n* | *%* |
|---|---|---|---|---|---|---|---|---|
| 0 | 9 | 29 | 27 | 87 | 23 | 77 | 20 | 65 |
| 1–9 | 8 | 26 | 3 | 9 | 4 | 14 | 7 | 24 |
| 10–40+ | 14 | 46 | 1 | 3 | 3 | 10 | 4 | 12 |
| Totals | 31 | (100) | 31 | (100) | 30 | (100) | 31 | (100) |

| *Spouse* | *On admission* *n* | *%* | *10 days* *n* | *%* | *2 months* *n* | *%* | *6 months* *n* | *%* |
|---|---|---|---|---|---|---|---|---|
| 0 | 18 | 58 | 22 | 71 | 20 | 65 | 18 | 58 |
| 1–9 | 4 | 13 | 3 | 10 | 5 | 17 | 4 | 13 |
| 10–40+ | 9 | 29 | 6 | 20 | 6 | 20 | 9 | 29 |
| Totals | 31 | (100) | 31 | (100) | 31 | (100) | 31 | (100) |

Patients' smoking habits on admission and at 6 months $t=4.939$ $p<0.001$

**Table 6**

**Medication after discharge (number of prescribed drugs)**

| | *10 days* *n* | *%* | *2 months* *n* | *%* | *6 months* *n* | *%* |
|---|---|---|---|---|---|---|
| None | 9 | 29 | 7 | 23 | 7 | 23 |
| Occasional (GTN) | 0 | 0 | 4 | 13 | 1 | 3 |
| 1 or 2 tablets daily | 12 | 39 | 9 | 29 | 16 | 53 |
| 3 or more | 10 | 32 | 11 | 36 | 6 | 20 |
| Totals | 31 | (100) | 31 | (100) | 30 | (100) |

**Table 7**

**Activity at 6 months**

| | *Work* *n* | *%* | *Leisure Activity* *n* | *%* | *Exercise* *n* | *%* | *Sexual Activity* *n* | *%* |
|---|---|---|---|---|---|---|---|---|
| Return to: | | | | | | | | |
| Normal | 16 | 52 | 17 | 55 | 14 | 45 | 16 | 57* |
| Moderate | 1 | 3 | 7 | 23 | 7 | 23 | 0 | 0 |
| Poor | 10 | 32 | 7 | 23 | 10 | 32 | 12 | 45 |
| Retired | 4 | 13 | NA | | NA | | NA | |
| Totals | 31 | (100) | 31 | (100) | 31 | (100) | 28 | (100) |

* Normal=no sex for some. NA=Not applicable

**Table 8**

**Correlation (Pearson product moment, 'r') with satisfactory outcome**

| | *Work* | *Leisure activity* | *Exercise* | *Sexual activity* |
|---|---|---|---|---|
| Few symptoms | 0.593 *** | 0.795 *** | 0.826 *** | 0.634 *** |
| Good GP support | 0.580 *** | 0.436 * | 0.408 * | 0.183 * |
| Social class | 0.542 * | 0.219 NS | 0.023 NS | 0.368 * |
| Not smoking | 0.358 * | 0.533 ** | 0.433 * | 0.358 * |
| Not overweight | 0.377 * | 0.217 NS | 0.491 ** | 0.005 NS |
| Not depressed | 0.309 NS | 0.646 *** | 0.641 *** | 0.283 NS |
| Cardiac damage | 0.081 NS | 0.341 NS | 0.122 NS | 0.331 NS |

Significance levels ***$p<0.001$ **$p<.01$ *$p<.05$

*(continued on page 119)*

### Do we tell our coronary patients enough? *(continued from page 108)*

whether he was working on admission r=0.425 ($p<0.01$), being overweight r=0.380 ($p<0.05$), and with a lack of G.P. support r=0.356 ($p<0.05$). The correlation between symptoms and cardiac damage was not significant r=0.103 (NS).

### CONCLUSIONS

The findings of this study support the work of others (16, 17, 18), that survival and return to work are not adequate measures of successful outcome, and that there is considerable distress among the survivors of myocardial infarction which is associated with social and psychological aspects of the illness. Symptoms which patients consider to be an indication of their medical condition are associated with social and psychological factors rather than measures of cardiac damage (Table 8).

The patients in this study were less well informed and had less understanding of their condition than they would have liked. A better understanding and more information and advice could lead to improvements in both the psychological and the somatic symptoms experienced. Perhaps not surprisingly patients who had good support from their family doctor and those who were not smoking had better outcomes than those who were poorly supported and continued to smoke.

### ACKNOWLEDGEMENT

We wish to thank the Bristol and Weston District Medical Research Committee for their financial support and the physicians and nursing staff of the Bristol Royal Infirmary for their co-operation throughout the study.

### REFERENCES

1. FINLAYSON, A., McEWEN, J. Coronary Heart Disease and Patterns of Living. London: Croom Helm, 1977.
2. MAGUIRE, P. Communication skills in patient care. In: Steptoe, A., Matthews, A., eds. Health Care and Human Behavior. London: Academic Press, 1984: 153–73.
3. JENKINS, B., MAYBERY, J. F., KENT, S., et al. Patients' evaluation of a post-myocardial infarction teaching programme. Post Grad Med J 1984; 60: 30–2.
4. LEY, P. Satisfaction, compliance and communication. Br J Clin Psychol 1982; 21: 241–54.
5. GEORGE, C. F., WATERS W. E., NICHOLAS, J. A. Prescription information leaflets: a pilot study in general practice. Br Med J 1983; 287: 1193–6.
6. CAY, E. The influence of psychological problems in returning to work after Myocardial Infarction. In: Chatterjee, D. S. and Parkes, H. G., eds. Cardiac Rehabilitation: Proceedings of a Society of Occupational Medicine Research Panel Symposium. 1983: 42–62.
7. BECKER, M., ROSENSTOCK, I. Compliance with medical care. In: Steptoe, A., Mathews, A., eds. Health Care and Human Behavior. London: Academic Press 1984: 175–208.
8. MASON, C., MURDOCK, J. C. Current opinion concerning the treatment of heart disease. J Roy Coll Gen Pract 1983; 33: 91–9.
9. MAYOU, R., WILLIAMSON, B., FOSTER, A. Attitudes and Advice after myocardial infarction. Br Med J 1976; 1: 1577–9.
10. SPEEDLING, E. Heart Attack. London: Tavistock, 1982.
11. NORRIS, R. N., BRANDT, P. W. T., CAUGHEY DE LEE, A. J., et al. A new prognostic index. Lancet 1969; 1: 274–8.
12. MERRILEES, M. A., SCOTT, P. J., NORRIS, R. M. Progress after a myocardial infarction: results of a 15 year follow up. Br Med J 1984; 288: 356–9.
13. BROWN, G., KANDINSKI, H. Rehabilitation Strategies After Myocardial Infarction: Preliminary Report from a Comparative Study in West Germany and the United Kingdom. University of Aston, Unpublished report, 1981.
14. GOLDBERG, D. P., COOPER, B., EASTWOOD M. R., et al. A standardised psychiatric interview for use in community surveys. Br J Prev Soc Med 1970; 24: 18–23.
15. TRELAWNY-ROSS, C. P., RUSSELL, O. Social and Psychological Responses to Myocardial Infarction. J Psychosom Res; in press.
16. MAYOU, R. The course and determinants of reaction to myocardial infarction. Br J Psychiat 1979; 134:588–94.
17. LLOYD, G. G., CAWLEY, R. H. Psychiatric morbidity after myocardial infarction. Q J Med 1982; 201: 33–42.
18. COLLING A. The rehabilitation of the coronary patient. In: Colling, A. ed. Coronary Care in the Community. Croom Helm: London, 1977: 201–210.

